# The Molecular Mechanism of Herpes Simplex Virus 1 UL31 in Antagonizing the Activity of IFN-β

**DOI:** 10.1128/spectrum.01883-21

**Published:** 2022-02-23

**Authors:** Lan Gong, Xiaowen Ou, Li Hu, Jiayi Zhong, Jingjing Li, Shenyu Deng, Bolin Li, Lingxia Pan, Liding Wang, Xuejun Hong, Wenqi Luo, Qiyuan Zeng, Jie Zan, Tao Peng, Mingsheng Cai, Meili Li

**Affiliations:** a State Key Laboratory of Respiratory Disease, The Second Affiliated Hospital, Guangdong Provincial Key Laboratory of Allergy & Clinical Immunology; Department of Pathogenic Biology and Immunology, Sino-French Hoffmann Institute, School of Basic Medical Sciences, Guangzhou Medical University, Guangzhou, Guangdong, China; b State Key Laboratory of Respiratory Disease, Sino-French Hoffmann Institute, School of Basic Medical Sciences, Guangzhou Medical University, Guangzhou, Guangdong, China; c Jinming Yu Academician Workstation of Oncology, Affiliated Hospital of Weifang Medical University, Weifang, Shandong, China; d School of Biomedical and Pharmaceutical Sciences, Guangdong University of Technology, Guangzhou, Guangdong, China; College of Veterinary Medicine, Oklahoma State University

**Keywords:** IFN-β, HSV-1, RLR, UL31, innate immunity

## Abstract

Virus infection triggers intricate signal cascade reactions to activate the host innate immunity, which leads to the production of type I interferon (IFN-I). Herpes simplex virus 1 (HSV-1), a human-restricted pathogen, is capable of encoding over 80 viral proteins, and several of them are involved in immune evasion to resist the host antiviral response through the IFN-I signaling pathway. Here, we determined that HSV-1 UL31, which is associated with nuclear matrix and is essential for the formation of viral nuclear egress complex, could inhibit retinoic acid-inducible gene I (RIG-I)-like receptor pathway-mediated interferon beta (IFN-β)–luciferase (Luc) and (PRDIII-I)4-Luc (an expression plasmid of IFN-β positive regulatory elements III and I) promoter activation, as well as the mRNA transcription of IFN-β and downstream interferon-stimulated genes (ISGs), such as ISG15, ISG54, ISG56, etc., to promote viral infection. UL31 was shown to restrain IFN-β activation at the interferon regulatory factor 3 (IRF3)/IRF7 level. Mechanically, UL31 was demonstrated to interact with TANK binding kinase 1 (TBK1), inducible IκB kinase (IKKi), and IRF3 to impede the formation of the IKKi-IRF3 complex but not the formation of the IRF7-related complex. UL31 could constrain the dimerization and nuclear translocation of IRF3. Although UL31 was associated with the CREB binding protein (CBP)/p300 coactivators, it could not efficiently hamper the formation of the CBP/p300-IRF3 complex. In addition, UL31 could facilitate the degradation of IKKi and IRF3 by mediating their K48-linked polyubiquitination. Taken together, these results illustrated that UL31 was able to suppress IFN-β activity by inhibiting the activation of IKKi and IRF3, which may contribute to the knowledge of a new immune evasion mechanism during HSV-1 infection.

**IMPORTANCE** The innate immune system is the first line of host defense against the invasion of pathogens. Among its mechanisms, IFN-I is an essential cytokine in the antiviral response, which can help the host eliminate a virus. HSV-1 is a double-stranded DNA virus that can cause herpes and establish a lifelong latent infection, due to its possession of multiple mechanisms to escape host innate immunity. In this study, we illustrate for the first time that the HSV-1-encoded UL31 protein has a negative regulatory effect on IFN-β production by blocking the dimerization and nuclear translocation of IRF3, as well as promoting the K48-linked polyubiquitination and degradation of both IKKi and IRF3. This study may be helpful for fully understanding the pathogenesis of HSV-1.

## INTRODUCTION

A variety of pathogens, including viruses, can trigger the cellular innate immune response, which is essential to limit the early spread of pathogens. After virus invades the host, the intracellular signal transmission requires a class of pattern recognition receptors (PRRs) to specifically recognize the pathogen-associated molecular patterns (PAMPs) (1). Retinoic acid-inducible gene I (RIG-I)-like receptors (RLRs), one of the essential types of PRRs, are a family of DExD/H box RNA helicases that function as cytoplasmic sensors of viral RNA. There are three members of the RLRs, including RIG-I, melanoma differentiation-associated factor 5 (MDA5), and laboratory of genetics and physiology 2 (LGP2) (2, 3). Both RIG-I and MDA5 have an N-terminal region consisting of a caspase recruitment domain (CARD), and by CARD-CARD interactions, they bind to the downstream mitochondrial antiviral signaling protein (MAVS) and then recruit tumor necrosis factor receptor-associated factor 3 (TRAF3) and TRAF6. In turn, TANK binding kinase 1 (TBK1)/inducible I kappa B kinase (IKKi) signaling cascades are activated, leading to the entrance of interferon (IFN) regulatory factor 3 (IRF3)/IRF7 and nuclear factor kappa B (NF-κB) into the nucleus to activate the transcription and expression of type I interferon (IFN-I), as well as the proinflammatory cytokines (4).

Herpesviruses are large, enveloped, linear double-stranded DNA (dsDNA) viruses, ranging in size from 120 to 260 nm. The virion is composed of four elements, including core, capsid, tegument, and envelope. Herpes simplex virus 1 (HSV-1) is a typical human herpesvirus that mainly leads to lip herpes, keratitis, and skin herpetic eczema, although serious cases can cause dangerous diseases like sporadic encephalitis. When HSV-1 from primary infection of epithelial or mucosal cells is transferred to the sensory ganglions, a lifelong latent infection is established. However, new lytic infection occurs in epithelial or mucosal cells when latent HSV-1 is reactivated (5).

The genome of HSV-1 is about 152 kb and encodes at least 84 viral proteins (6). Recent studies show that HSV-1 has developed diverse mechanisms to antagonize the IFN-I response. US11, an RNA binding tegument protein (7), interacts with RIG-I and MDA5 through its C-terminal domain, thereby suppressing the formation of RIG-I/MAVS and MDA5/MAVS complexes, which leads to decreases in IRF3 activity and IFN-β production (8). γ_1_34.5, a multifunctional neurovirulence factor (9, 10), disrupts the interaction between TBK1 and IRF3, which inhibits the phosphorylation and nuclear translocation of IRF3, as well as its subsequent induction of IFN and IFN-stimulated genes (ISGs), and thus helps virus replication and neural invasion (11, 12). US3, a Ser/Thr kinase, can interact with and hyperphosphorylate IRF3 at Ser175 to prevent the dimerization and nuclear translocation of IRF3 (13). Moreover, US3 can also act as an inhibitor of NF-κB signaling by reducing the polyubiquitination of TRAF6 through its kinase activity and inhibiting Toll-like receptor 2 (TLR2) signaling at the early stage of viral infection (14). Tegument protein VP16 remarkably impedes Sendai virus (SeV)-induced interferon beta (IFN-β) production by selectively interfering with IRF3 to block recruitment of its coactivator CREB binding protein (CBP) for the formation of the transcriptional complex IRF3-CBP (15). ICP27, the conserved herpesvirus protein, can interact with TBK1 and STING by targeting the TBK1-activated STING signalosome, which finally inhibits the expression of IFN-I in human macrophages (16).

UL31 protein (306 amino acids), the HSV-1-encoded gene product of *UL31* that contains a hydrophilic amino terminus and a nuclear localization signal (17), is thought to be conserved in all members of the *Herpesviridae* family (18). UL31 is a phosphorylated protein that acts on the formation of the viral nucleocapsid envelope at the initial stage. It can change the localization of nuclear membrane components and destroy the nuclear membrane to advance the primary envelope acquisition and nuclear leakage of the nucleocapsid (19–21). It is reported that UL31 can interact with UL34 to form a heterodimeric complex that localizes at the inner nuclear membrane (INM) in HSV-1-infected cells (22). This protein complex can disrupt the nuclear lamina and facilitate the access of herpesvirus nucleocapsid to the INM and the acquisition of a primary viral envelope, which is critical for viral nuclear egress (19, 23). UL31 may also help to anchor virus products and promote the synthesis and packaging of viral DNA (24). However, whether UL31 is involved in the regulation of IFN-I production and its molecular mechanism in the antiviral immune response are still unknown.

In this study, we found that UL31 could inhibit the activation of the RLR signaling pathway-induced IFN-β promoter and downregulate the expression of ISGs (ISG15, ISG54, ISG56, OAS1, and STAT1). By interacting with TBK1, IKKi, and IRF3, UL31 could inhibit the formation of the IKKi-IRF3 complex and then block SeV-induced IRF3 dimerization and nuclear translocation. However, UL31 did not inhibit the formation of CBP/p300-IRF3 complex. Furthermore, UL31 was able to promote K48-linked polyubiquitination of both IKKi and IRF3, leading to their proteasome-mediated degradation, which eventually resulted in the downregulation of IFN-β activity.

## RESULTS

### UL31 inhibits SeV-mediated IFN-β activation and transcription of downstream ISGs.

**FIG 1:**
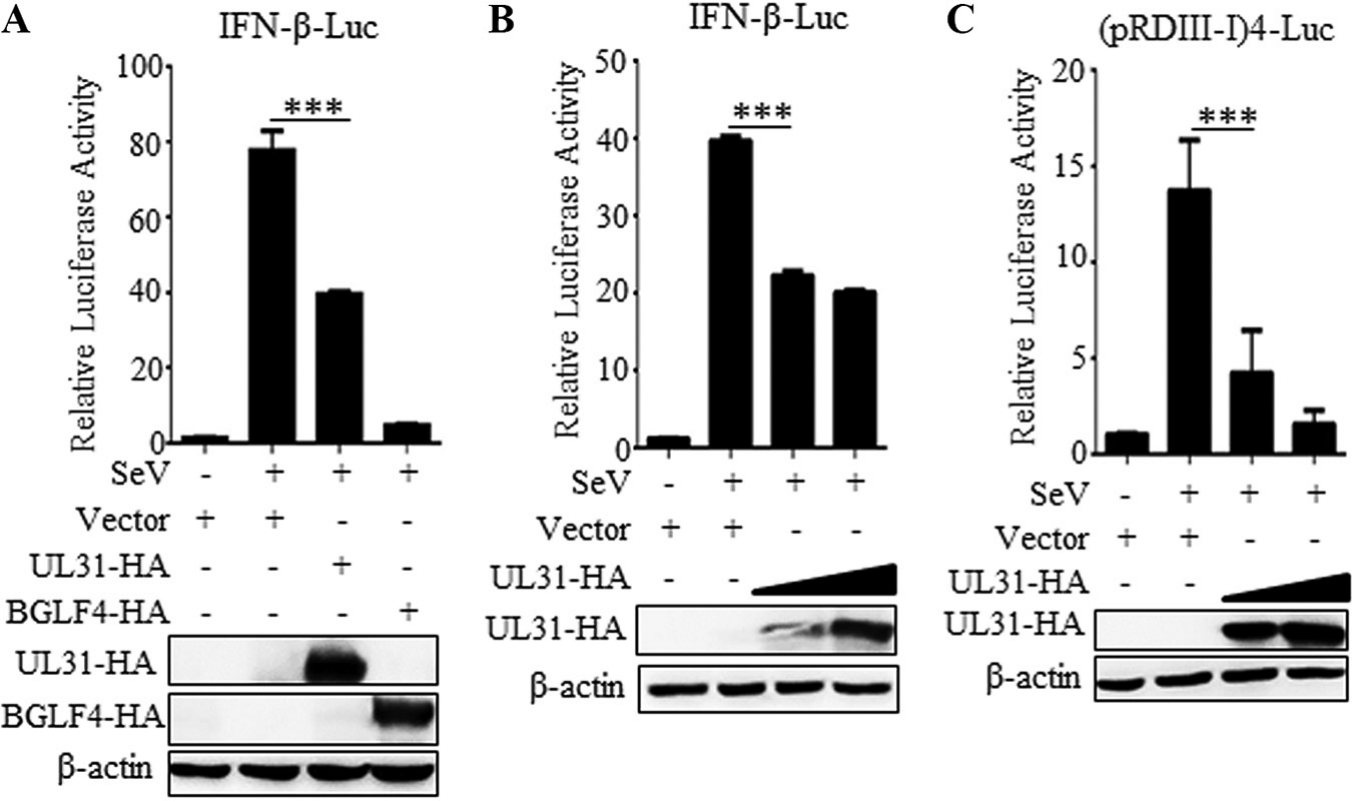
HSV-1 UL31 inhibits the SeV-mediated activation of IFN-β. (A) HEK293T cells were transfected with IFN-β-Luc (100 ng), RL-TK-Luc (10 ng) and empty vector, pUL31-HA-N1 (500 ng) or pBGLF4-HA (500 ng) expression plasmid. (B–C) IFN-β-Luc (100 ng) (B) or (pRDIII-I)4-Luc (100 ng) (C) was co-transfected with RL-TK-Luc (10 ng) and increased amounts (500, 1000 ng) of UL31-HA expression plasmid into HEK293T cells. At 24 h post-transfection, cells were infected with or without 100 HAU/ml of SeV. At 16 h post-infection, IFN-β-driven luciferase activity was determined by DLR. Statistical analysis was performed using the Student's t test. ***, *P* < 0.001.

In order to analyze the function of HSV-1 UL31 in the regulation of virus-mediated activation of IFN-β, empty vector or a UL31-hemagglutinin (HA) expression plasmid was coexpressed with IFN-β–luciferase (Luc) reporter into HEK293T cells, and then the cells were infected with SeV and dual-luciferase reporter (DLR) assay was performed. Compared to the results for mock infection, the IFN-β–Luc promoter activity was induced by a fold of ∼80 after SeV stimulation for 16 h, but this activity was obviously inhibited in the presence of UL31 (Fig. 1A), similar to the inhibitory effect of the positive control BGLF4 (25). Additionally, UL31 restrained IFN-β promoter activity in a dose-dependent manner (Fig. 1B).

As an important cytokine, IFN-β is mainly regulated by IRF3, NF-κB, and other transcription factors (26, 27). To illustrate whether UL31 constrains the activation of IRF3, empty vector or the UL31-HA expression plasmid was cotransfected with the (PRDIII-I)4-Luc promoter expression plasmid (an expression plasmid of IFN-β positive regulatory elements III and I) into HEK293T cells, and cells were then infected with SeV. It was clearly seen that SeV could induce evident promoter activity of (PRDIII-I)4-Luc, but this activity was inhibited when UL31 was overexpressed in the cells (Fig. 1C), suggesting that UL31 can suppress IFN-β activation through the IRF3 branch.

**FIG 2:**
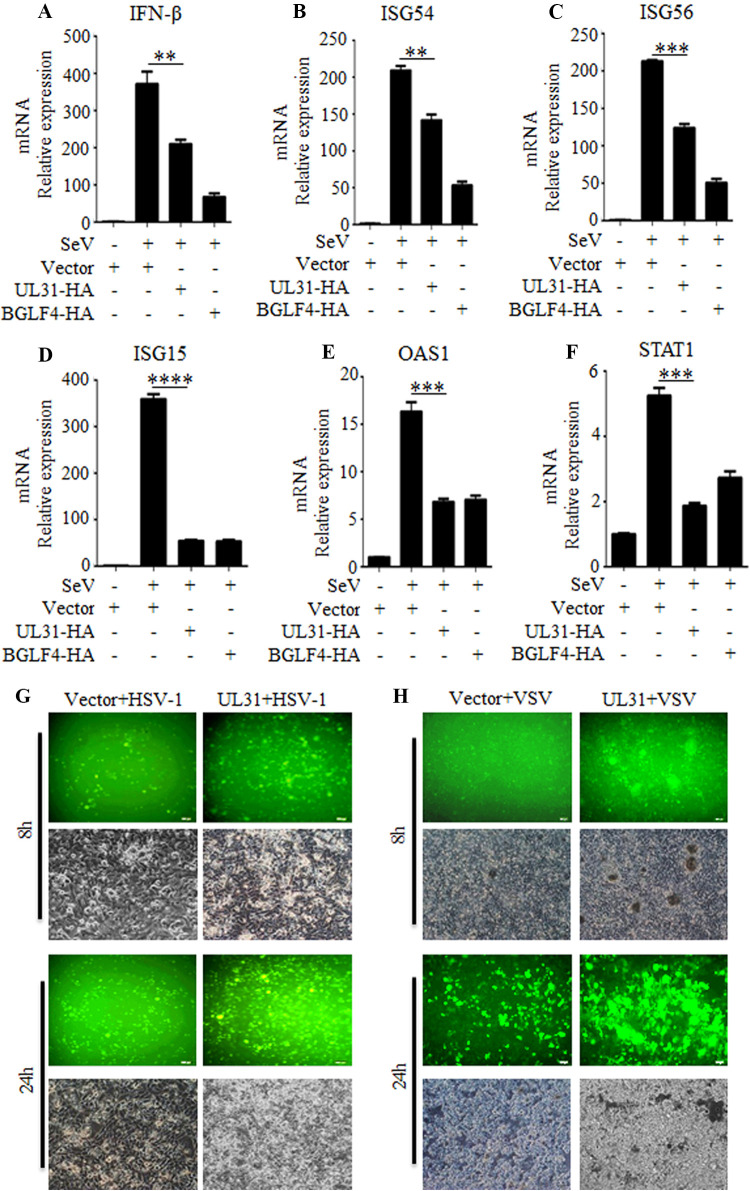
HSV-1 UL31 downregulates the mRNA expression of SeV-mediated IFN-β and downstream ISGs to facilitate the replication of DNA virus and RNA virus. (A to F) HEK293T cells were transfected with empty vector, UL31-HA, or BGLF4-HA expression plasmid. At 24 h posttransfection, cells were mock infected or infected with 100 HAU/mL of SeV for 16 h, and then total RNA was extracted and subjected to real-time qPCR with primers for IFN-β, ISG15, ISG54, ISG56, OAS1, STAT1, and GAPDH. The expression levels of target genes were normalized with that of the control GAPDH, and the relative fold value is the ratio of the expression value in each reaction mixture to the value for vector-transfected cells. Statistical analysis was performed using Student’s *t* test. The data represent mean values ± SD for 3 replicates. **, *P < *0.01; ***, *P < *0.001; ****, *P < *0.0001. (G, H) Empty vector or UL31-HA expression plasmid was transfected into HEK293T cells for 24 h, and then cells were infected with GFP-tagged HSV-1 (G) or GFP-tagged VSV (H) at an MOI of 1. An IFN sensitivity assay was performed to test the promoting effects of UL31 on HSV-1 and VSV replication at the indicated times, and the replication of these two kinds of viruses was analyzed using a fluorescence microscope.

IFN-I is a crucial cytokine that exerts its biological effects through the induction of hundreds of ISGs, and the expression profiles of these ISGs vary with different viruses (27). To verify the results mentioned above, HEK293T cells were transfected with a UL31-HA or a BGLF4-HA expression plasmid or empty vector and infected with SeV. The results showed that the overexpression of UL31 could repress not only the mRNA transcription of IFN-β (Fig. 2A) but also the downstream ISGs, such as ISG15, ISG54, ISG56, OAS1, and STAT1 (Fig. 2B to F), confirming that UL31 is capable of suppressing the activity of IFN-β and downregulating the transcription of downstream ISGs.

### UL31 negatively regulates the cellular antiviral immune response.

To further define the role of UL31 in inhibiting IFN-β production and the host antiviral immune response, infection experiments using different viruses were carried out. HEK293T cells were transfected with empty vector or UL31-HA expression plasmid and then infected with green fluorescent protein (GFP)-tagged HSV-1 (DNA virus) or vesicular stomatitis virus (VSV) (RNA virus). At different times after infection, the replication of HSV-1 and VSV was analyzed by fluorescence microscopy. Compared with the results for the empty vector-transfected control, overexpression of UL31-HA could induce a more obvious cytopathic effect and produce more virus fluorescence when cells were infected with HSV-1 (Fig. 2G) or VSV (Fig. 2H), and the number of fluorescent cells in the visual field increased continuously. Taken together, the results showed that UL31 can downregulate the host antiviral immune response, thus promoting the proliferation of different viruses.

### UL31 may restrain the IFN-β signaling pathway at the level of IRF3/IRF7.

**FIG 3:**
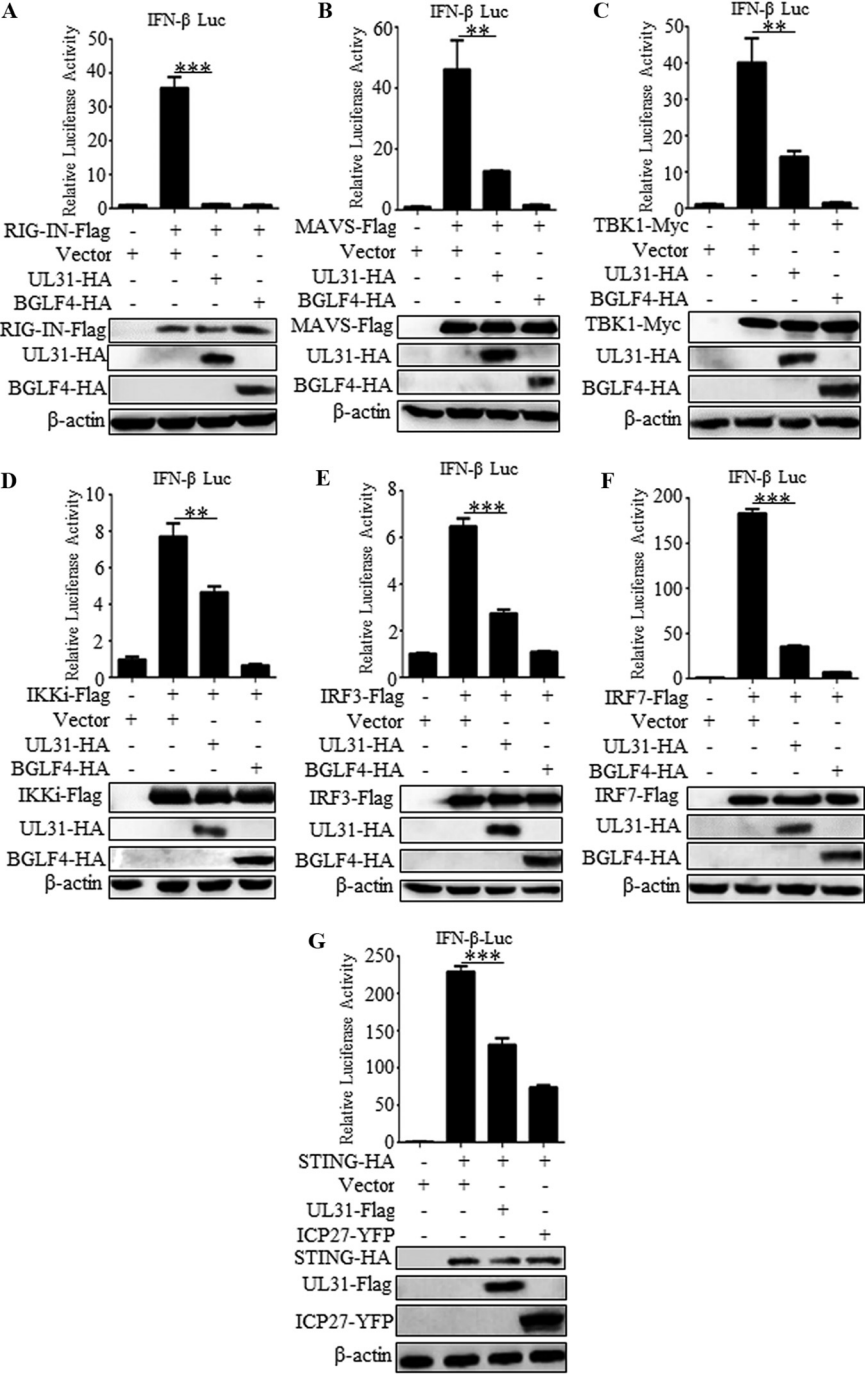
HSV-1 UL31 inhibits the promoter activation of IFN-β at the IRF3/IRF7 level. HEK293T cells were transfected with plasmid expressing RIG-IN-Flag (A), MAVS-Flag (B), TBK1-Myc (C), IKKi-Flag (D), IRF3-Flag (E), IRF7-Flag (F), or STING-HA (G), together with pIFN-β-Luc, pRL-TK, and empty vector or the UL31-HA, BGLF4-HA, or ICP27-EYFP expression plasmid. At 24 h posttransfection, luciferase assays were performed. Data are expressed as the fold induction, and the expression of all adaptor proteins, including RIG-IN, MAVS, TBK1, IKKi, IRF3, IRF7, and STING, was confirmed by WB. The data represent mean values ± SD for 3 replicates. Statistical analysis was performed using Student’s *t* test. **, *P* < 0.01; ***, *P* < 0.001.

In the RLR pathway, several innate immune adaptor proteins license IRF3 activation to induce IFN (28), which also happens in the cyclic GMP-AMP synthase (cGAS)-STING (stimulator of interferon gene) signaling pathway induction of IFN (29). To determine at what level UL31 blocks the expression of IFN-β, expression plasmids of UL31-HA and each of the RLR signaling pathway components, including RIG-IN (active form of RIG-I), MAVS, TBK1, IKKi, IRF3, and IRF7, were cotransfected into HEK293T cells. As shown by the results in Fig. 3, all expression constructs of the signaling pathway components resulted in a 10- to 200-fold induction of the IFN-β-Luc reporter activity, and the activation driven by RIG-IN, MAVS, TBK1, IKKi, IRF3, and IRF7 was conspicuously inhibited in the presence of UL31 (Fig. 3A to F), indicating that UL31 may inhibit the RLR signaling pathway at the level of IRF3/IRF7. In addition, UL31 also hampered STING-triggered IFN-β-Luc reporter activity when compared with the positive-control ICP27 (Fig. 3G) (16), suggesting that UL31 may negatively regulate the cGAS-STING signaling pathway.

### UL31 interacts with TBK1, IKKi, and IRF3.

**FIG 4:**
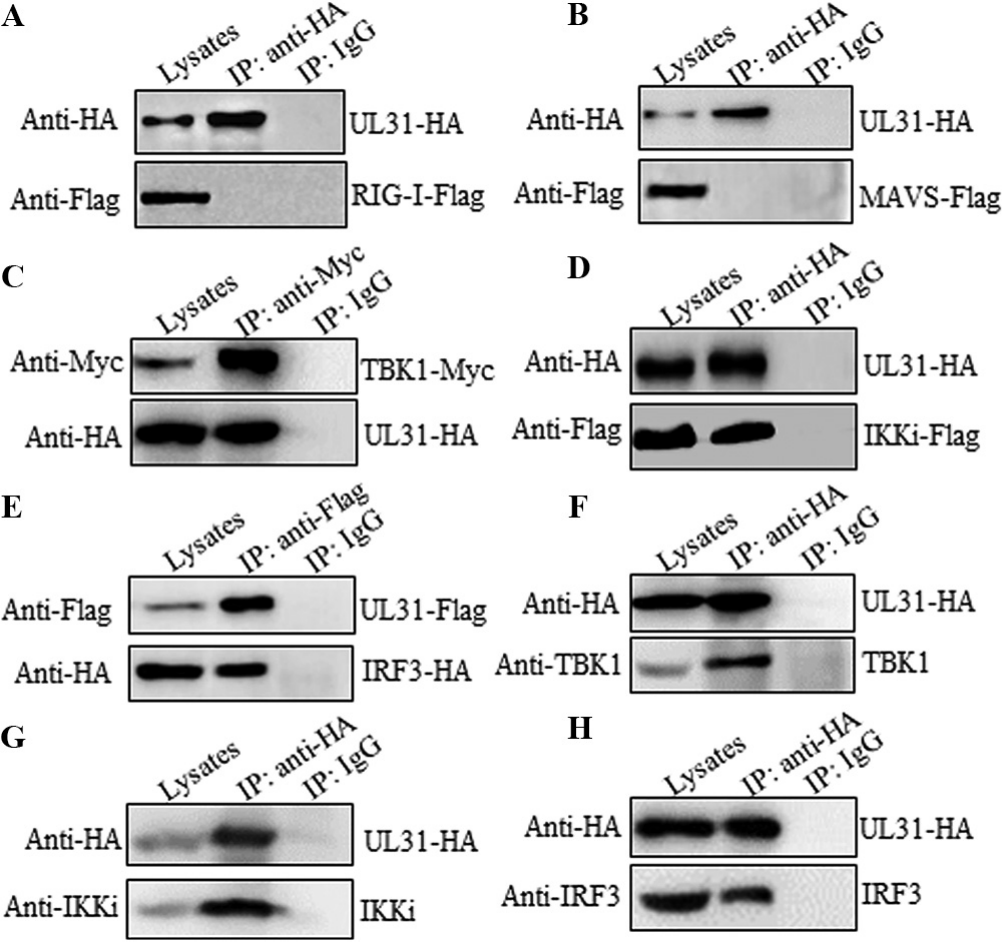
HSV-1 UL31 interacts with overexpressed and endogenous TBK1, IKKi, and IRF3. (A to E) An expression plasmid combination of UL31-HA/RIG-I-Flag (A), UL31-HA/MAVS-Flag (B), UL31-HA/TBK1-Myc (C), UL31-HA/IKKi-Flag (D), or UL31-Flag/IRF3-HA (E) was cotransfected into HEK293T cells. (F to H) The UL31-HA expression plasmid was transfected into HEK293T cells. At 24 h posttransfection, cells were harvested and lysed. The samples were then subjected to Co-IP using mouse anti-HA (A, B, D, and F to H), anti-Myc (C), or anti-Flag (E) MAb or nonspecific IgG. Immunoprecipitated samples were then separated by 10% SDS–PAGE, and proteins were transferred onto NC membranes. WB was performed with the indicated antibodies. Rabbit anti-TBK1 MAb, anti-IKKi MAb, and anti-IRF3 MAb were used to detect the expression of endogenous TBK1 (F), IKKi (G), and IRF3 (H), respectively.

The results described above showed that UL31 may destroy the IFN-β signaling pathway at the level of IRF3/IRF7. Therefore, we investigated the potential interaction between UL31 and adapter proteins. Expression plasmid combinations expressing UL31-HA/RIG-I–Flag, UL31-HA/MAVS-Flag, UL31-HA/TBK1-Myc, UL31-HA/IKKi-Flag, and UL31-Flag/IRF3-HA were cotransfected into HEK293T cells, and coimmunoprecipitation (Co-IP) was performed with anti-HA, anti-Myc, or anti-Flag monoclonal antibody (MAb). As the results showed that overexpressed RIG-I or MAVS could not be immunoprecipitated with UL31 (Fig. 4A and B), UL31 itself could therefore act as a negative-control protein here. However, overexpressed UL31 was obviously immunoprecipitated with TBK1 (Fig. 4C), and overexpressed IKKi and IRF3 were also immunoprecipitated with UL31 (Fig. 4D and E). To continue to dissect whether UL31 can interact with endogenous adaptors, the expression plasmid of UL31-HA was transfected into HEK293T cells and Co-IP was performed with anti-HA MAb. The results showed that UL31 interacted with endogenous TBK1 (Fig. 4F), IKKi (Fig. 4G), and IRF3 (Fig. 4H). Based on the above-described results, UL31 can interact not only with IRF3 but also with TBK1 and IKKi.

### UL31 inhibits the formation of the IKKi-IRF3 complex.

**FIG 5:**
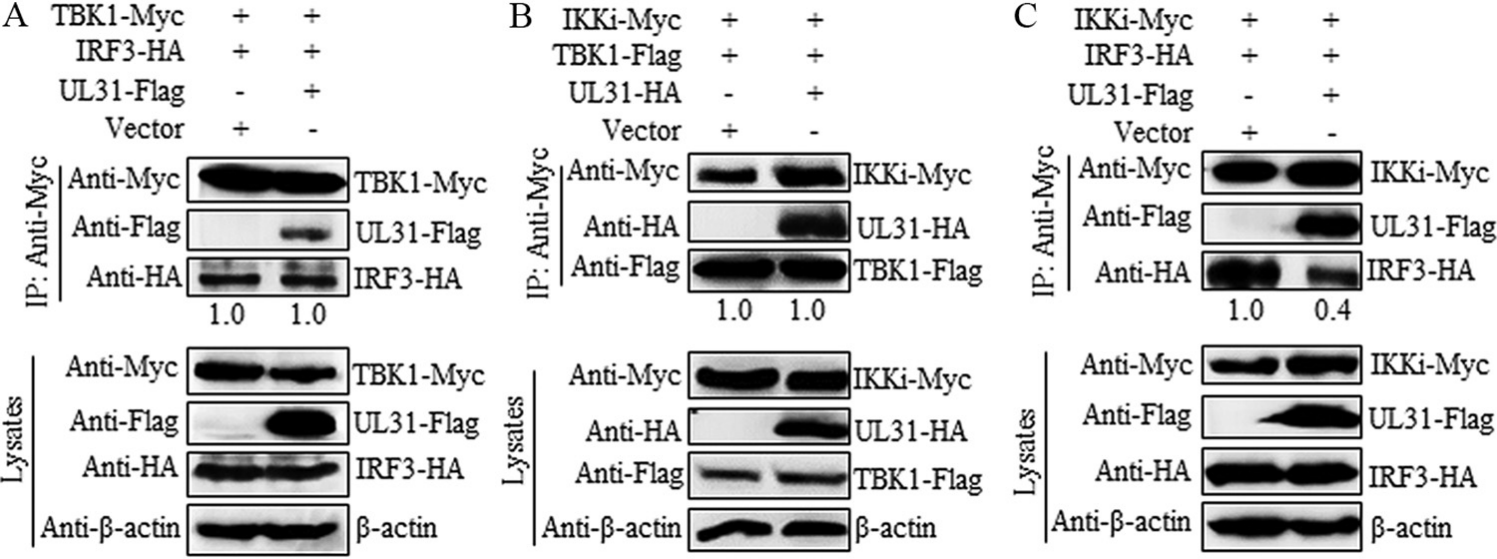
HSV-1 UL31 impedes the formation of the IKKi-IRF3 complex. Empty vector or UL31 expression plasmid (UL31-Flag or UL31-HA) was cotransfected with the expression plasmid combination of TBK1-Myc/IRF3-HA (A), TBK1-Flag/IKKi-Myc (B), or IKKi-Myc/IRF3-HA (C) into HEK293T cells for 24 h. Cells were then harvested and analyzed by Co-IP using mouse anti-Myc MAb. WB was performed with anti-Flag, anti-HA, anti-Myc, and anti-β-actin MAbs, and the blots were analyzed using Image J.

After the activation of RIG-I in the IFN-I signaling pathway, the downstream adaptor protein MAVS is activated, which in turn recruits and activates TBK1 and IKKi kinases to activate the structurally closed IRF3 (28). To further analyze whether UL31 can affect the interactions among TBK1, IKKi, and IRF3, empty vector or the UL31 expression plasmid (UL31-Flag or UL31-HA) was cotransfected with the expression plasmid combination of TBK1-Myc/IRF3-HA, IKKi-Myc/TBK1-Flag, or IKKi-Myc/IRF3-HA into HEK293T cells, and then Co-IP was performed with anti-Myc MAb. The results showed that UL31 could not inhibit the interaction of TBK1/IRF3 (Fig. 5A) or IKKi/TBK1 (Fig. 5B), but it could distinctly restrain the formation of the IKKi-IRF3 complex (Fig. 5C).

### UL31 interacts with IRF7 but cannot affect the formation of IRF7-related complexes.

**FIG 6:**
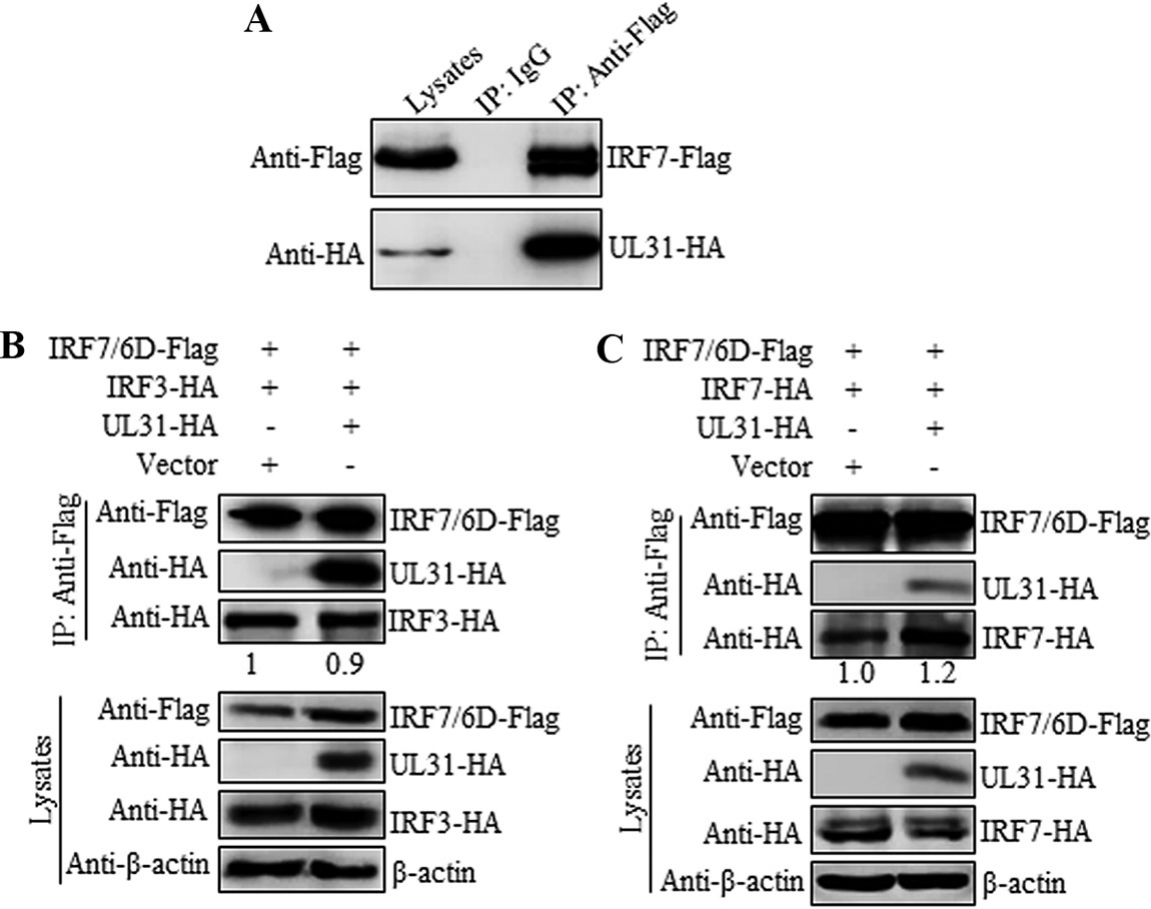
HSV-1 UL31 binds to IRF7 but cannot disturb the formation of IRF7-related complexes. (A) HEK293T cells were cotransfected with UL31-HA and IRF7-Flag expression plasmids. (B, C) Empty vector or UL31-HA expression plasmid was cotransfected with the expression plasmid combination of IRF3-HA/IRF7-6D-Flag (B) or IRF7-HA/IRF7-6D-Flag (C) into HEK293T cells for 24 h. Cells were then harvested and analyzed by Co-IP using mouse anti-Flag MAb. WB was performed with anti-Flag, anti-HA, and anti-β-actin MAbs, and the blots were analyzed by Image J.

In addition to IRF3, IRF7 is also a critical transcription factor in the innate immune response and can form the heterodimer IRF3/IRF7 or homodimer IRF7/IRF7 to activate the downstream production of IFN-I (30). Therefore, we investigated the potential interaction between UL31 and IRF7. HEK293T cells were cotransfected with UL31-HA and IRF7-Flag expression plasmids, and then Co-IP was performed with anti-Flag MAb. The results showed that UL31 was notably immunoprecipitated with IRF7 (Fig. 6A). Since UL31 could interact with IRF7, we next tested whether UL31 can impact the formation of IRF7-related complexes. Empty vector or the UL31-HA expression plasmid was cotransfected with expression plasmid combination of IRF3-HA/IRF7-6D-Flag or IRF7-HA/IRF7-6D-Flag into HEK293T cells, and then Co-IP was carried out with anti-Flag MAb. It was shown that UL31 could not inhibit the interaction of IRF3/IRF7-6D (Fig. 6B) or IRF7/IRF7-6D (Fig. 6C), suggesting that UL31 cannot attenuate the formation of IRF7-related complexes.

### UL31 cannot disrupt TBK1 activation but can restrict IRF3 activation.

**FIG 7:**
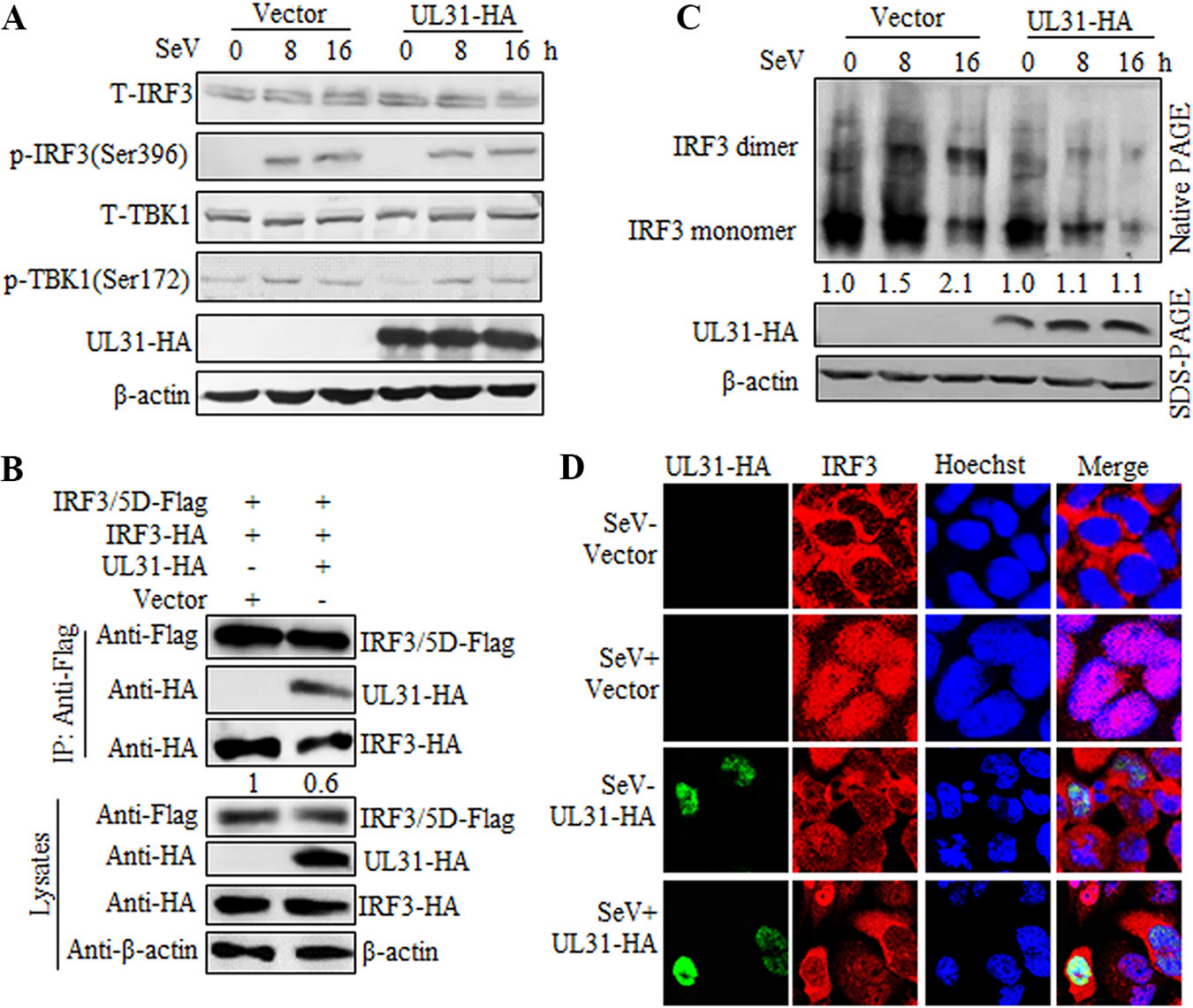
HSV-1 UL31 blocks IRF3 dimerization and nuclear trafficking. (A) Empty vector or UL31-HA expression plasmid was transfected into HEK293T cells for 24 h, and then cells were mock infected or infected with 100 HAU/mL SeV and harvested at the indicated times (0, 8, and 16 h). Cells were lysed, and the supernatant was subjected to SDS-PAGE and probed with anti-IRF3 MAb, phospho-(p-)IRF3 (Ser396), anti-TBK1 MAb, p-TBK1 (Ser172), anti-HA MAb, or anti-β-actin MAb. Here, T-IRF3 indicates total IRF3, and T-TBK1 indicates total TBK1. (B) Empty vector or UL31-HA expression plasmid was cotransfected with the expression plasmid combination of IRF3-HA/IRF3-5D-Flag into HEK293T cells for 24 h. Cells were then harvested and analyzed by Co-IP using mouse anti-Flag MAb. WB was performed with anti-Flag, anti-HA, and anti-β-actin MAbs. (C) Empty vector or UL31-HA expression plasmid was transfected into HEK293T cells for 24 h, and then cells were mock infected or infected with 100 HAU/mL SeV and harvested at the indicated times (0, 8, and 16 h). Cells were lysed, and whole-cell extracts were subjected to native PAGE and probed with anti-IRF3 MAb to detect IRF3 monomer and dimer. Anti-HA and anti-β-actin MAbs were used to detect the expression of UL31 and β-actin, respectively. The blots were analyzed using Image J. (D) HeLa cells were transfected with empty vector or UL31-HA expression plasmid. At 24 h posttransfection, cells were mock infected or infected with 100 HAU/mL SeV for 8 h. Then, cells were stained with primary antibodies mouse anti-HA MAb and rabbit anti-IRF3 MAb. Subsequently, FITC-conjugated goat anti-mouse IgG (green) and Cy5-conjugated goat anti-rabbit IgG (red) were used as the secondary antibodies. Cell nuclei were stained with Hoechst 33342 (blue). The images were obtained by laser scanning confocal microscopy (SP8; Leica Microsystems).

In the antiviral immune response, IRF3 can be phosphorylated at multiple serine and threonine residues by upstream kinases TBK1 and IKKi (31). Meanwhile, TBK1 also can be activated by autophosphorylation at residue Ser172 (32). To examine whether UL31 can affect SeV-mediated phosphorylation of IRF3 and TBK1, HEK293T cells were transfected with either empty vector or UL31-HA expression plasmid, and then cells were infected with SeV for the times indicated below. After infection for 8 h, SeV could induce the phosphorylation of IRF3 Ser396, and this was obviously enhanced when the infection reached 16 h. However, this phosphorylation was not influenced when UL31 was overexpressed in cells (Fig. 7A). Similarly, the level of TBK1 phosphorylation at Ser172 was increased with infection time, but this phosphorylation was not affected at all when UL31 was overexpressed in cells (Fig. 7A).

IRF3 dimerization was also detected in the presence or absence of UL31. Empty vector or the UL31-HA expression plasmid was cotransfected with the expression plasmid combination of IRF3-5D-Flag/IRF3-HA into HEK293T cells, and then Co-IP was carried out with anti-Flag MAb. It was demonstrated that UL31 could restrain the interaction of IRF3-5D/IRF3 (Fig. 7B). In addition, when HEK293T cells were transfected with either empty vector or the UL31-HA expression plasmid and infected with SeV for the indicated times, SeV could induce marked IRF3 dimerization with the prolongation of infection time, and this dimerization was reduced when UL31 was overexpressed in the cells (Fig. 7C).

Upon phosphorylation and dimerization, IRF3 will transfer from the cytoplasm to the nucleus and combine with the CBP/p300 coactivators to promote the production of IFN-β (15). As the aforementioned results indicated that UL31 could repress SeV-induced IRF3 dimerization, we continued to probe whether UL31 can perturb the nuclear translocation of IRF3. When HeLa cells were not stimulated with SeV, IRF3 localized exclusively in the cytoplasm, and it transferred from the cytoplasm to the nucleus after SeV infection. However, a certain proportion of the SeV-mediated nuclear translocation of IRF3 could be inhibited in the presence of UL31 (Fig. 7D). Taken together, UL31 can not abolish TBK1 or IRF3 phosphorylation, but it can restrain IRF3 dimerization and nuclear accumulation.

### UL31 interacts with p300/CBP but does not inhibit the formation of the p300/CBP-IRF3 complex.

**FIG 8:**
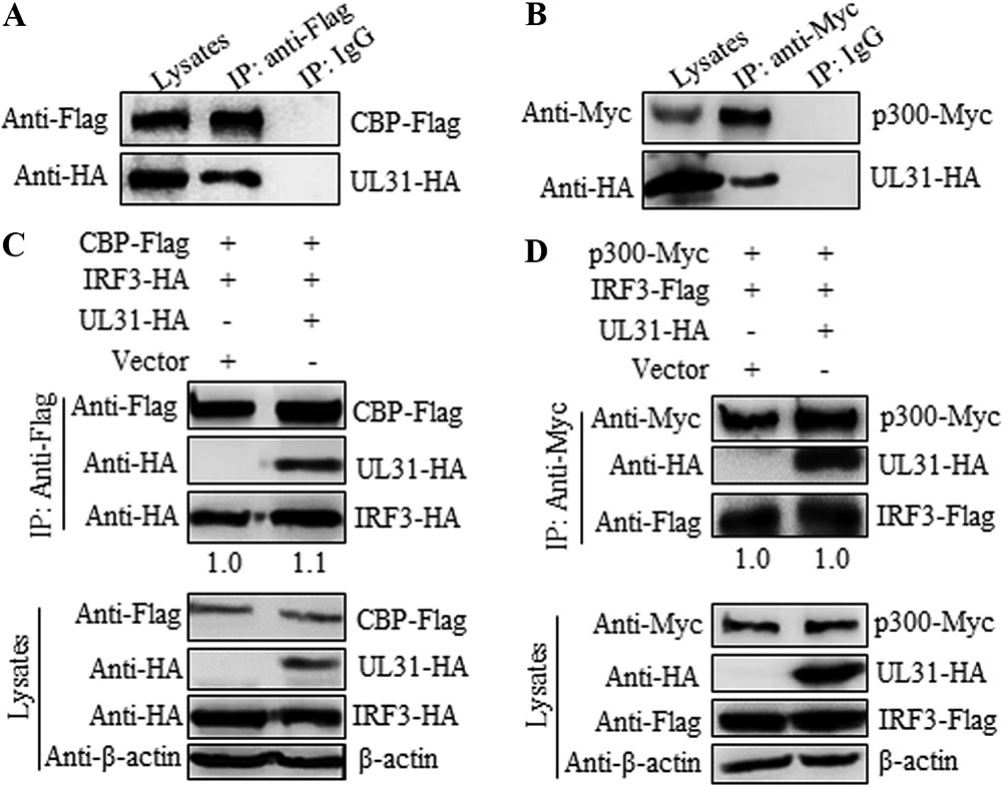
HSV-1 UL31 binds to p300/CBP but does not interfere with the formation of the CBP/p300-IRF3 complex. (A, B) HEK293T cells were cotransfected with the expression plasmid combination of UL31-HA/CBP-Flag (A) or UL31-HA/p300-Myc (B). (C, D) Empty vector or the UL31-HA expression plasmid was cotransfected with the expression plasmid combination of IRF3-HA/CBP-Flag (C) or IRF3-Flag/p300-Myc (D) into HEK293T cells. At 24 h posttransfection, cells were harvested and lysed. The samples were then subjected to Co-IP using mouse anti-Flag MAb (A and C), anti-Myc MAb (B and D), or nonspecific IgG. WB was performed with anti-HA, anti-Flag, anti-Myc, and anti-β-actin MAbs, and the blots were analyzed using Image J.

As transcription-costimulatory molecules of IRF3, histone acetyltransferases p300/CBP can acetylate the nucleosome and bind to activated IRF3 dimers, thus effectively initiating gene expression of IFN (33, 34). Since UL31 could block the nuclear translocation of IRF3, we continued to explore the potential interaction between UL31 and p300 or CBP. HEK293T cells were cotransfected with expression plasmids of UL31-HA and CBP-Flag or p300-Myc, and then Co-IP was performed with anti-Flag or anti-Myc MAb. The results showed that UL31 was associated with both CBP and p300 (Fig. 8A and B). Therefore, we wondered whether UL31 can interfere with the formation of the CBP-IRF3 or p300-IRF3 complex. Empty vector or UL31-HA expression plasmid was cotransfected with the expression plasmid combination of CBP-Flag/IRF3-HA or p300-Myc/IRF3-Flag into HEK293T cells, and then Co-IP was performed with anti-Flag or anti-Myc MAb. It was seen that neither the CBP and IRF3 interaction (Fig. 8C) nor the p300 and IRF3 interaction (Fig. 8D) was inhibited when UL31 was overexpressed in cells.

### UL31 accelerates IKKi and IRF3 degradation by mediating their K48-linked polyubiquitinations.

**FIG 9:**
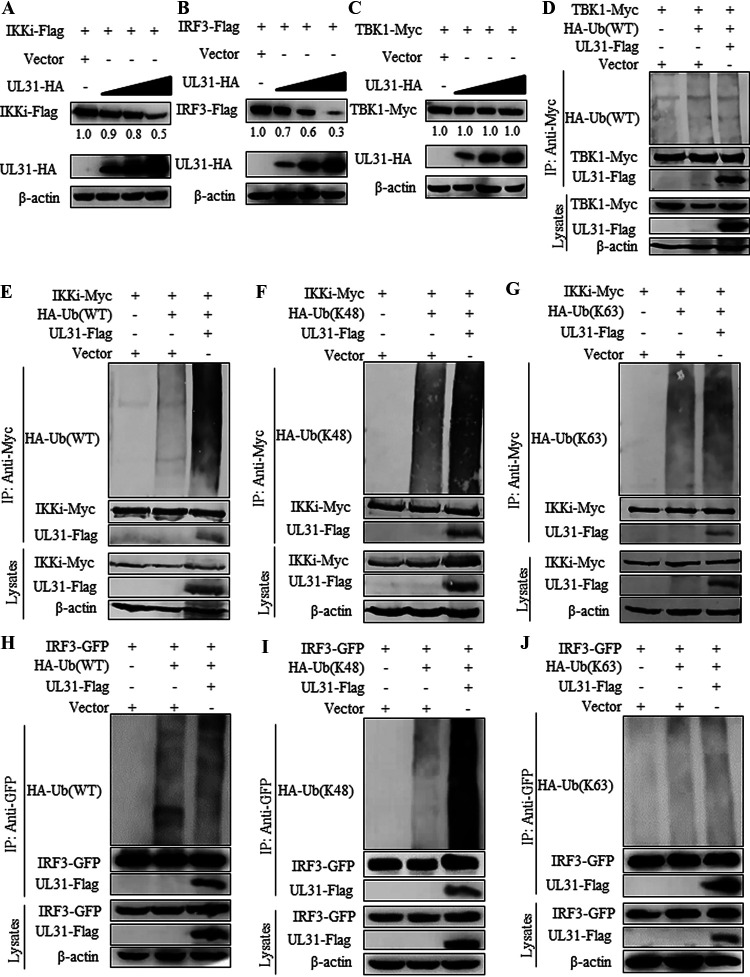
HSV-1 UL31 advances K48-linked-polyubiquitination degradation of IKKi and IRF3. (A to C) An amount of 500 ng of IKKi-Flag (A), IRF3-Flag (B), or TBK1-Myc (C) expression plasmid was cotransfected with increasing amounts (500, 1,000, and 1,500 ng) of UL31-HA expression plasmid into HEK293T cells for 24 h. Cells were then lysed, and the supernatant was subjected to WB analysis and probed with mouse anti-Myc, anti-Flag, anti-HA, or anti-β-actin MAb. (D) Empty vector or UL31-Flag expression plasmid was cotransfected with TBK1-Myc into HEK293T cells, along with WT-Ub-HA expression plasmid. (E to G) Empty vector or UL31-Flag expression plasmid was cotransfected with IKKi-Myc into HEK293T cells, along with WT-Ub-HA (E), K48-Ub-HA (F) or K63-Ub-HA (G) expression plasmid. (H to J) Empty vector or UL31-Flag expression plasmid was cotransfected with IRF3-GFP into HEK293T cells, along with WT-Ub-HA (H), K48-Ub-HA (I), or K63-Ub-HA (J) expression plasmid. At 24 h posttransfection, cells were lysed, and the supernatant was immunoprecipitated using anti-Myc (D to G) or anti-GFP (H to J) MAb, followed by WB analysis using anti-HA, anti-Flag, anti-Myc, anti-GFP, and anti-β-actin MAbs. The blots were analyzed by Image J.

Viral proteins can mediate the degradation of important adaptor proteins, causing the activity of adaptor proteins to be affected to allow the virus to escape the innate immune response (35). Since UL31 could bind to TBK1, IKKi, and IRF3, we next examined whether UL31 can impact their expression. When the expression plasmid of TBK1-Myc, IKKi-Flag, or IRF3-Flag was cotransfected with increasing amounts of the UL31-HA expression plasmid into HEK293T cells, UL31 could induce protein degradation of IKKi and IRF3 in a dose-dependent manner (Fig. 9A and B), but it had no influence on the expression of TBK1 (Fig. 9C).

Since K48-linked polyubiquitination usually results in the degradation of the target protein (36), we speculated whether UL31 regulates IFN-β expression by the K48-linked polyubiquitination of IKKi and/or IRF3. A TBK1-Myc, IKKi-Myc, or IRF3-GFP expression plasmid was cotransfected with an HA-Ub (wild type [WT]), HA-Ub (K48), or HA-Ub (K63) ubiquitin-related expression plasmid into HEK293T cells, with or without UL31-Flag. Then, Co-IP was performed, using anti-Myc or anti-GFP MAb. The results demonstrated that UL31 could not affect the ubiquitination of TBK1 (Fig. 9D), which is consistent with the above-described result that UL31 was unable to degrade TBK1. Surprisingly, UL31 could promote the polyubiquitination of IKKi and IRF3 with the expression of HA-Ub (WT) and HA-Ub (K48), but not HA-Ub (K63) (Fig. 9E to J). These results suggested that UL31 led to the degradation of IKKi and IRF3 through the proteasome pathway. In short, UL31 can catalyze the K48-linked polyubiquitination and degradation of IKKi and IRF3 and therefore negatively regulate the IFN signaling pathway.

## DISCUSSION

HSV-1 UL31 is a multifunctional protein. It is phosphorylated in infected cells, and it also partitions with the nuclear matrix (37). The expression of UL31 and UL34 at the nuclear membrane is necessary for alphaherpesviruses to form an effective nuclear egress complex, while UL31 becomes especially susceptible to proteasomal degradation in the absence of UL34 (38). It has been previously described that UL31, UL34, and US3 localize on the nuclear membrane and perinuclear virions (20), and this colocalization can be regulated by UL31 in HSV-1-infected cells via phosphorylating US3 or by a US3-independent mechanism (39). γ_1_34.5 targets cellular p32 and protein kinase C to bridge the UL31/UL34 complex through UL31, which facilitates the nucleocapsid in transiting to the cytoplasm to promote the release and growth of virus (40). Similarly, the homologues of UL31, such as Kaposi’s sarcoma-associated herpesvirus (KSHV) open reading frame 69 (ORF69), human cytomegalovirus (HCMV) UL53, and Epstein-Barr virus (EBV) BFLF2, need to interact with viral proteins to perform their functions. ORF69 interacts with KSHV ORF67 and induces membrane proliferation to remodel these membranes into circular-virion-sized vesicles (41). UL53 forms patches at the nuclear periphery and colocalizes with lamin B in a cytoplasmic perinuclear granular formation (42). BFLF2 colocalizes with BFRF1 in vesicles derived from the nuclear margin and cytoplasmic nuclear membrane in coexpressing cells (43). In the present study, we found that HSV-1 UL31 can inhibit the production of IFN-β, which provides a potentially new function for UL31 and other herpesvirus orthologs in innate immunity.

It has been shown that UL31 can activate the NF-κB signaling pathway to promote the expression of the immediate early protein ICP4, early protein ICP8, and late protein glycoprotein C (gC) for the efficient infection of HSV-1 (44). Furthermore, UL31 also plays an important role in the synthesis, cleavage, and packaging of HSV-1 DNA. UL31 can advance the envelopment of the nucleocapsid during its transport from the nucleus into the cytoplasm, and the yield of infectious progeny virions can diminish dramatically, by 1,000 to 10,000-fold, when UL31 is deleted (24). Moreover, HSV-1 can inhibit IFN-I production through various proteins (45, 46), and distinct virus proteins may have synergistic or compensative effects on suppressing IFN-I production. During HSV-1 infection, the inhibition of IFN-I conducted by certain viral proteins may be compensated by other viral proteins when these proteins are knocked out. Therefore, the inhibitory result of wild-type HSV-1 on IFN-I production may not be notably different from that of HSV-1 with the gene encoding one of these proteins knocked out.

As a consequence of the dissection of the activities of UL31 described above, we consider that the obstructive potency of HSV-1 on IFN-I production may not alter noticeably when UL31 is knocked out. Despite the fact that the suppression of IFN-I production by HSV-1 is weakened when UL31 is knocked out, it may not be straightforwardly caused by the decrease in UL31-mediated IFN-I production due to UL31 knockout; rather, it may be provoked by the evident defect of HSV-1 replication following UL31 deletion, leading to serious reduction of virus titers, which also decreases the HSV-1-prompted inhibition of IFN-I, since many other HSV-1 proteins that can interfere with the production of IFN-I are also reduced. Accordingly, it is difficult to distinguish whether the diminished inhibition of IFN-I production during HSV-1 infection is the direct consequence of UL31 deletion. That is to say, the UL31-mediated interruption of IFN-I production is a challenge to be investigated in HSV-1-infected physiological cells.

The IFN-I signaling pathway is an important component of innate immunity, comprised of IFN-α and IFN-β. After stimulation by virus, the IFN-induced ISGs are activated and mediate a broad range of immune responses to limit the propagation of HSV-1 infection (47). The results of our study showed that UL31 acts as a negative regulatory factor in the SeV-induced IFN-β pathway. It inhibits the activation of the IFN-β promoter at the IRF3/IRF7 level and interacts with TBK1, IKKi, and IRF3, which finally restrains the transcription activation of IFN-β and downstream ISGs. Moreover, UL31 plays a role in increasing early gene expression during early HSV-1 infection, so as to optimize viral protein production and the activation of Jun N-terminal protein kinase (JNK) and NF-κB in a manner independent of the transactivator ICP27 (44). It is well known that the early activation of NF-κB may promote the efficient replication of the virus (48), and thus, HSV-1 takes advantage of UL31 to activate NF-κB for initiating viral gene transcription in the early stage of viral infection, while HSV-1 again exploits UL31 to inhibit IFN-β for suppressing the host immune response and facilitating virus proliferation in the late stage of viral infection.

IRF3 is the most crucial transcription factor that mediates the expression of IFN-I. It contains a conserved N-terminal DNA-binding domain and a C-terminal IRF association domain (IAD) that mediates phosphorylation and homodimerization or heterodimerization with other IRF members and interacts with CBP/p300 (49, 50). The homologous proteins IKKi and TBK1 of IκB kinase are kinases upstream from IRF3 that directly phosphorylate the C-terminal domain of IRF3 after virus invasion, resulting in the activation of IRF3 (51, 52). Although TBK1 is well documented to take a major role in the yield of IFN-I (51, 53, 54), the generation of IFN-I is not affected in TBK1-impaired macrophages (54). It has been reported that the arenavirus nucleoprotein can colocalize with IKKi and associate with its kinase domain to prohibit its autocatalytic activity and IRF3-regulated signaling (55). Dengue virus NS2B/3 protease can bind to IKKi and hinder the phosphorylation (Ser386) and nuclear trafficking of IRF3 to lessen the secretion of IFN-I (56). The results of our previous study show that the EBV early lytic protein BFRF1 can specifically target IKKi (but not TBK1) and disturb its kinase activity, which finally impedes the RLR-induced phosphorylation, dimerization, and nuclear translocation of IRF3 (57). Collectively, these studies suggest that IKKi does not play a redundant role in activating IRF3 and that it is also indispensable for facilitating the production of IFN-I. Consequently, the contributions of TBK1 and IKKi reveal that IRF3 phosphorylation and IRF3 signaling in cells are relevant to a sophisticated requirement of both kinases (55). In this study, we found that UL31 can inhibit the formation of IKKi-IRF3 complex. However, UL31 does not block the phosphorylation of IRF3 (Ser396) or TBK1 (Ser 172), but it does restrain the dimerization and nuclear translocation of IRF3.

As mentioned above, both TBK1 and IKKi are important in the RLR pathway for the signal transmission to induce IFN-I production. The possible reason why UL31 can inhibit the interaction of IKKi and IRF3 but not impede IRF3 phosphorylation (Ser396) is that the region where UL31 interacts with IKKi and IRF3 is not the key region in which IKKi phosphorylates IRF3 (Ser396) but the nuclear localization signal region of IRF3 and/or the key region for the formation of the IKKi-IRF3 complex. Therefore, UL31 may not have an effect on the phosphorylation of IRF3 (Ser396), but it can influence the formation of the IKKi-IRF3 complex and the nuclear accumulation of IRF3. Upon viral infection, cellular TBK1- and IKKi-mediated Ser385 and Ser386 phosphorylation of IRF3 and the Ser/Thr cluster between amino acids 396 and 405 of IRF3 leads to its conformational change and activation (52, 58, 59). Thus, UL31 may inhibit IRF3 phosphorylation at other residues but not Ser396. For example, dengue virus NS2B/3 protease can inhibit the phosphorylation of IRF3 at Ser386, and thus nuclear translocation, but not at Ser396 (56).

The transcriptional coactivators CBP and p300 are critical regulators of gene expression. A compactly folded 46-residue domain in CBP and p300 can bind to the C-terminal domain of IRF3, and then a holocomplex of IRF3 competent in DNA binding for full activation is generated (60, 61). Eventually, this complex binds to PRD I/III in the IFN-β promoter for transcription activation and IFN-β production (62). Human argonaute 2 (AGO2) is reported to interact with IRF3 by its MID domain when cells are infected with H5N1 virus, which does not block the phosphorylation, nuclear translocation, or DNA binding ability of IRF3, but it can inhibit its association with CBP to negatively regulate the IFN-I signaling pathway (63). In this study, we found that UL31 can interact with CBP/p300, but it cannot inhibit the formation of the transcription complex CBP/p300-IRF3, suggesting that UL31 may not inhibit the function of CBP/p300 in the production of IFN-I. Similarly, ICP0 does not affect the phosphorylation and dimerization of IRF3, but it colocalizes with activated IRF3 and CBP/p300 in the nucleus to inactivate IRF3 and accelerate its degradation, leading to decreased IFN-β transcription (64).

In addition to the phosphorylation of kinases and regulation of nuclear transcriptional coactivators, polyubiquitination is also an essential modification in the regulation of the IFN pathway. As one of the most studied types of polyubiquitination, K48-linked ubiquitin chains are originally described as the signal that targets substrates for proteasomal degradation, which is utilized for normal protein turnover in cells (36, 65). Nonclassical linkage types, such as K63-, K11-, and M1-linked chains, are instead associated with DNA repair regulation, cell cycle progression, innate immunity, and inflammation (66, 67). Several kinases and IRFs have been demonstrated to regulate the IFN pathway through various forms of polyubiquitination. For example, Raul, an E3 ligase containing the Hect domain, has been shown to promote the polyubiquitination and proteasome degradation of inactive IRF3 and IRF7 (68). TRIM26, a member of the tripartite motif (TRIM) protein family, composed of more than 70 members in humans, binds to IRF3 and transfers to the nucleus after virus infection to advance the K48-linked-polyubiquitin degradation of IRF3 in the nucleus (35). UBE2S, a member of the ubiquitin-conjugating enzyme family, acts as a negative regulator in the IFN signaling pathway, which interacts with TBK1 and recruits ubiquitin-specific protease 15 (USP15) to remove K63-linked polyubiquitin chains of TBK1 (69). Studies also show that some viral proteins can induce regulation of ubiquitination. The N terminus of UL36, the largest tegument protein of HSV-1, contains a deubiquitinase (DUB) motif (also known as UL36 ubiquitin-specific protease [UL36USP]) (70, 71) that can deubiquitinate TRAF3 to reduce its activity and diminish the recruitment of downstream TBK1, thereby preventing the production of IFN-β. Furthermore, the influenza A virus-encoded NS1 protein can inhibit IFN-I production by blocking E3 ubiquitin ligase TRIM25-mediated K63-linked polyubiquitination of the viral RNA sensor RIG-I (72). In our study, we found that HSV-1 UL31 promotes the K48-linked polyubiquitination of IKKi and IRF3 and targets their degradation through the proteasome pathway. Since IRF3 plays a key role in the signal pathway, its degradation inhibits its own protein activity, thereby impeding the production of downstream IFN-I.

Although some proteins (including viral proteins) can mediate the degradation of host proteins through a proteasome-dependent pathway, it may be necessary for the expression of the viral protein to reach a significantly higher level than that of the host protein to execute this function. It has been shown that TRAF3-interacting protein 3 (TRAF3IP3) can markedly inhibit RLR pathway-activated IFN-I production by triggering K48-linked-polyubiquitination degradation of TBK1. In the lysates of a TBK1 and TRAF3IP3 expression plasmids cotransfection and Co-IP experiment, TRAF3IP3 does not induce the degradation of TBK1 when their protein expression levels are comparable. However, TRAF3IP3 exhibits dose-dependent degradation of TBK1 when its protein expression level is remarkably higher than that of TBK1 (73). Therefore, it is reasonable that UL31 can induce K48-linked-polyubiquitination degradation of IKKi and IRF3 only when its protein expression level is markedly higher than those of IKKi and IRF3. As mentioned above, it is difficult to distinguish whether the ubiquitination modification of TBK1, IKKi, and/or IRF3 is directly triggered by UL31 when UL31 knockout HSV-1 and wild-type HSV-1 are used, since the UL31-mediated inhibition of IFN-I cannot be analyzed in HSV-1-infected physiological cells. Besides, it is possible that UL31 only affects the ubiquitination modification and degradation of IKKi and not that of TBK1, since IKKi is also equally as vital as TBK1.

**FIG 10:**
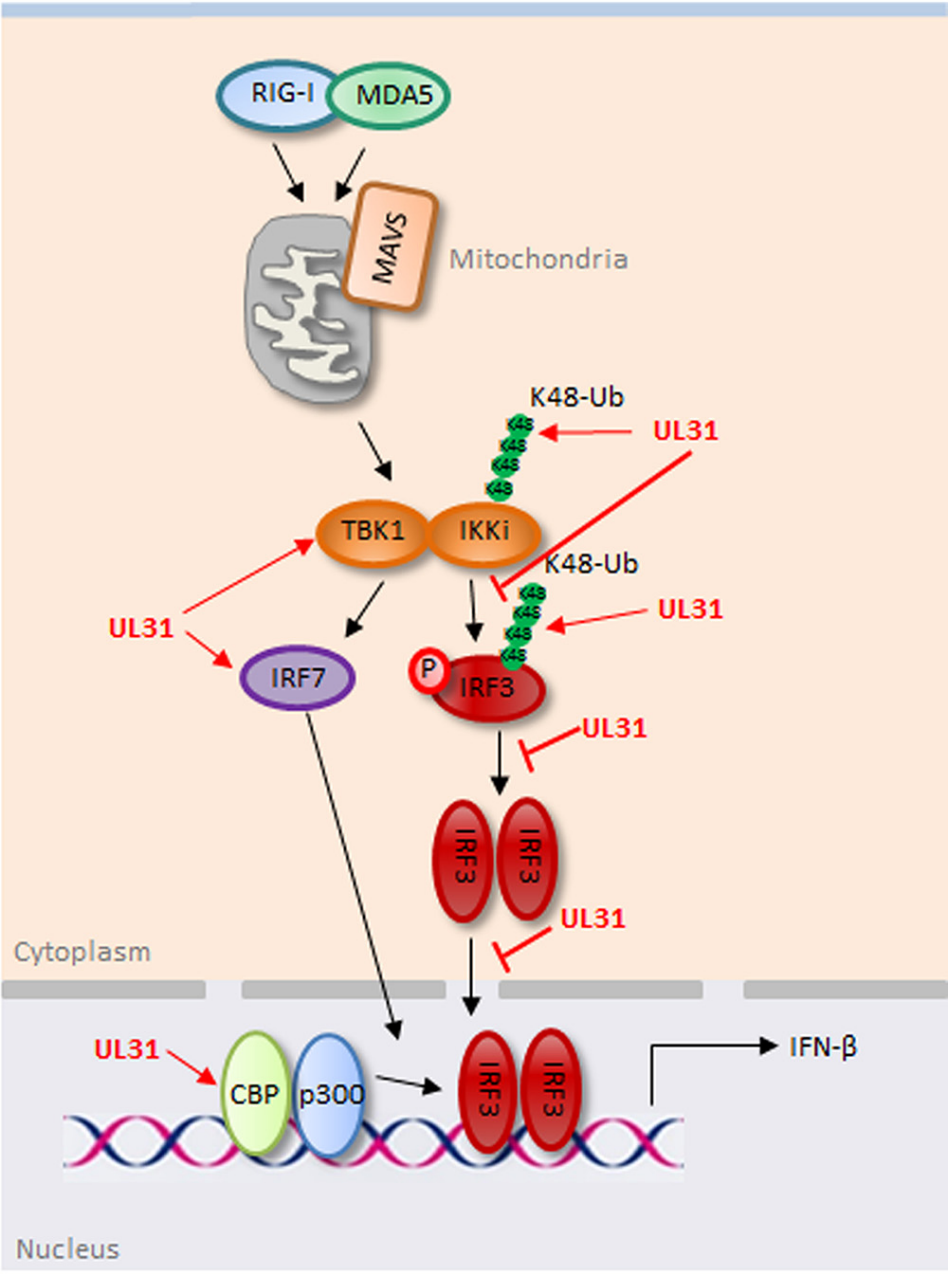
Schematic diagram of HSV-1 UL31 inhibiting the RLR-mediated IFN-β signaling pathway. In the RLR pathway, when the host is stimulated with virus, RIG-I and MDA5 bind to MAVS, and downstream kinases TBK1 and IKKi are recruited to activate IRF3 and/or IRF7 for the induction of IFN-β. In this study, HSV-1 UL31 could interact with TBK1, IKKi, and IRF3 to inhibit the formation of the IKKi-IRF3 complex, but it could not affect the formation of IRF7-related complexes. Moreover, UL31 could restrain the dimerization and nuclear translocation of IRF3. However, UL31 could not inhibit the formation of the p300/CBP-IRF3 complex in the nucleus. By accelerating the K48-linked polyubiquitination of IKKi and IRF3, UL31 led to the degradation of IKKi and IRF3 through the proteasome pathway, which finally played a negative role in the regulation of IFN-β production.

In summary, this study showed that HSV-1 UL31 inhibits IFN-β promoter activity at the level of IRF3/IRF7 and downregulates the expression of related ISGs. UL31 can block the formation of the IKKi-IRF3 complex by interacting with TBK1, IKKi, and IRF3. Moreover, UL31 restrains the dimerization and nuclear translocation of IRF3, thereby reducing the activity of IRF3. In addition, UL31 can promote the K48-linked polyubiquitination of IKKi and IRF3 to mediate their proteasome degradation, which ultimately downregulates the activity of IFN-β (Fig. 10). Our study revealed for the first time the regulatory role of HSV-1 UL31 in the RLR signaling pathway, which uncovers a new mechanism for HSV-1 to escape the host’s innate immunity, providing new ideas for future research on viral pathogenesis and vaccine development.

## MATERIALS AND METHODS

### Cells and antibodies.

Human embryonic kidney 293T (HEK293T) cells, HeLa cells, COS-7 cells, and Vero cells were grown in Dulbecco's modified Eagle medium (DMEM; Thermo Fisher Scientific, Waltham, MA, USA) supplemented with 10% heat-inactivated fetal bovine serum (FBS) and 100 U/mL of penicillin and streptomycin at 37°C in a 5% CO_2_ incubator. Mouse anti-Flag, anti-Myc, anti-GFP, and anti-hemagglutinin (HA) monoclonal antibodies (MAbs) were acquired from Abmart (Berkeley Heights, NJ, USA). IgG negative-control antibody was purchased from Proteintech (Wuhan, China). Rabbit anti-β-actin MAb was bought from ABclonal (Wuhan, China). Rabbit anti-IKKi, anti-TBK1, anti-IRF3, anti-phosphorylated (phospho)-IRF3 (Ser396), and anti-phospho-TBK1 (Ser172) MAbs, alkaline phosphatase (AP)-conjugated goat anti-mouse IgG and goat anti-rabbit IgG, and horseradish peroxidase (HRP)-conjugated goat anti-mouse IgG and goat anti-rabbit IgG were provided by Cell Signaling Technology (Boston, MA, USA). Cyanine 5 (Cy5)-conjugated goat anti-rabbit IgG and fluorescein isothiocyanate (FITC)-conjugated goat anti-mouse IgG were supplied by Bioss (Beijing China) and BBI Life Sciences, respectively.

### Virus strains.

SeV and GFP-tagged vesicular stomatitis virus (VSV-GFP) were preserved in our laboratory. The wild-type (WT) F strain of HSV-1 bacterial artificial chromosome (BAC) GFP Luc (simultaneously expressing firefly luciferase and GFP tags) was provided by Chunfu Zheng (Fujian Medical University) (74) and was propagated in Vero cells and preserved in our laboratory.

### Plasmid construction.

All enzymes used for cloning were from Thermo Fisher Scientific, and T4 DNA ligase was provided by TaKaRa (Kyoto, Japan), except for DNA polymerase KOD-plus-Neo, which was from Toyobo (Osaka, Japan). The UL31 ORF (918 bp) was amplified by PCR from the genomic DNA of HSV-1 F strain (HSV-1 BAC GFP Luc) using the following primers: UL31-F (5′-CGA AGC TTC GGA ATT CAT GTA TGA CAC CGA CCC CCA TC-3′, sense) and UL31-R (5′-GCA AGC TTA GGA TCC GTC GGC GGA GGA AAC TCG TCG AA-3′, antisense). As described in our previous studies, the purified fragment was treated with EcoRI and BamHI and ligated into the likewise-digested vectors pHA-N1 (regenerated from pEYFP-N1, Clontech) and pCMV-Flag (regenerated from pFlag-CMV-2, Sigma) to produce expression plasmids pUL31-HA and pUL31-Flag, respectively. Reporter plasmids pIFN-β-Luc (John Hiscott), (PRDIII-I)4-Luc (Stephan Ludwig), and NF-κB-Luc and pRL-TK (expressing firefly luciferase and *Renilla* luciferase, respectively; Zhengli Shi), as well as the expression plasmids pIKKi-Flag (Rongtuan Lin), pRIG-IN-Flag, RIG-I-Flag, STING-HA, pCBP-Flag, pp300-Myc, pIRF3-GFP, pIRF7-Flag, pICP27-enhanced yellow fluorescent protein (EYFP) (HSV-1), HA-Ub (WT), HA-Ub (K48), and HA-Ub (K63) (Chunfu Zheng), pMAVS-Flag, pIKKi-Myc, and pTBK1-Flag (Jun Cui), pIRF3-HA (Takemasa Sakaguchi), and pIRF3-Flag, pIRF3/5D-Flag, and pIRF7/6D-Flag (Yiling Lin) were kindly donated by the indicated individuals. The BGLF4-HA expression plasmid was created in our previous study (75).

### Transfection and dual luciferase reporter assays.

Dual luciferase reporter (DLR) assays were performed as previously described (57, 75–77). HEK293T cells were plated on 24-well plates and cotransfected with 100 ng of firefly Luc reporter plasmid [pIFN-β-Luc or (PRDIII-I)4-Luc], 10 ng of pRL-TK (to normalize transfection efficiency), and the indicated expression plasmids mixed with polyethylenimine transfection reagent according to the manufacturer’s instructions. At 24 h posttransfection, cells were lysed and luciferase assays were performed with a luciferase assay kit (Promega, Madison, WI).

### RNA isolation and quantitative PCR.

The reverse transcription quantitative PCR (qPCR) was performed according to our previous studies (57, 75, 76, 78). At 24 h posttransfection of the indicated plasmid(s), HEK293T cells were infected with or without 100 hemagglutinating units (HAU)/mL SeV for 16 h. Then, cells were lysed and total RNA was extracted with TRIzol (Thermo Fisher Scientific) according to the manufacturer's guidelines. The quantification of gene transcripts was performed by real-time qPCR using the Sybr green procedure and the CFX96 real-time PCR detection system (Bio-Rad, Hercules, CA, USA). The reverse-transcribed cDNA was denatured at 95°C for 1 min and amplified with 40 cycles of denaturation at 95°C for 10 s and annealing at 56°C for 25 s, followed by extension at 72°C for 20 s and 80°C for 10 s. The expression values of target genes were normalized to the value for the glyceraldehyde 3-phosphate dehydrogenase (GAPDH) control. The relative fold expression was the ratio of the expression value in each reaction mixture to the value for vector-transfected cells. The primer sets used for each gene of interest were as follows: for GAPDH, 5′-CAT CAT CCC TGC CTC TAC TG-3′ (sense) and 5′-GCC TGC TTC ACC ACC TTC-3′ (antisense) (79); for IFN-β, 5′-CAT CCC TGA GGA GAT TAA GC-3′ (sense) and 5′-AGA CAT TAG CCA GGA GGT TC-3′ (antisense); for ISG15, 5′-TGG ACA AAT GCG ACG AAC CTC-3′ (sense) and 5′-TCA GCC GTA CCT CGT AGG TG-3′ (antisense); for ISG54, 5′-GGA GGG AGA AAA CTC CTT GGA-3′ (sense) and 5′-GGC CAG TAG GTT GCA CAT TGT-3′ (antisense); for ISG56, 5′-AAG GCA GGC TGT CCG CTT A-3′ (sense) and 5′-TCC TGT CCT TCA TCC TGA AGC T-3′ (antisense) (15); for OAS1, 5′-AGC TTC GTA CTG AGT TCG CTC-3′ (sense) and 5′-CCA GTC AAC TGA CCC AGG G-3′ (antisense); and for STAT1, 5′-CGGCTGAATTTCGGCACCT-3′ (sense) and 5′-CAGTAACGATGAGAGGACCCT-3′ (antisense).

### Viral infection.

For detecting viral replication after disrupting the IFN-β production mediated by HSV-1 UL31, HEK293T cells were cultured on 6-well plates and transfected with vector or the UL31 expression plasmid. At 24 h posttransfection, the medium was exchanged for FBS-free medium containing HSV-1 or VSV at a multiplicity of infection (MOI) of 1. The virus was incubated with cells for 2 h and replaced with medium supplemented with 2% FBS to continue the incubation for the indicated times, and then cells were examined with fluorescence microscopy to detect the GFP fluorescence.

### Western blot analysis.

Western blot (WB) analysis was performed as previously described (80–83). Briefly, whole-cell extracts were subjected to 10% SDS–polyacrylamide gel electrophoresis (PAGE) and transferred to polyvinylidene difluoride (PVDF) or nitrocellulose (NC) membrane, followed by blocking with 5% nonfat milk in Tris-buffered saline (TBS), and then probed with the indicated primary antibodies overnight at 4°C. After washing with TBS-Tween 20 (TBST), the membrane was incubated with HRP-conjugated goat anti-rabbit IgG or goat anti-mouse IgG or with AP-conjugated goat anti-mouse IgG or goat anti-rabbit IgG. Then, an enhanced chemiluminescence assay or AP color development was carried out to display specific protein bands.

### Co-IP assay.

The coimmunoprecipitation (Co-IP) assay was performed in line with previous studies (80, 83–85). In short, HEK293T cells were cotransfected with 10 μg of each of the indicated expression plasmids. At 24 h posttransfection, cells were harvested and lysed on ice with 1,000 μL of lysis buffer. For each immunoprecipitation (IP), an aliquot of lysate was incubated with 10 μg of mouse anti-Myc, anti-HA, anti-Flag, or anti-GFP MAb or nonspecific IgG and 30 μL of a 1:1 slurry of protein A/G plus–agarose (Santa Cruz Biotechnology, Dallas, TX, USA) for at least 4 h or overnight at 4°C. The protein-bound beads were washed 4 times with 1,000 μL of lysis buffer containing 500 mM NaCl and then subjected to WB analysis.

### Native PAGE.

A native PAGE experiment was executed as described in previous studies (15, 57). HEK293T cells were transfected with empty vector or the UL31-HA expression plasmid. At 24 h posttransfection, cells were mock infected or infected with 100 HAU/mL SeV for 0, 8, or 16 h. Then, whole-cell extracts were subjected to native PAGE analysis. In brief, the gel was prerun with 25 mM Tris and 192 mM glycine (pH 8.4), with 1% deoxycholate (DOC) in the cathode chamber for 30 min at 40 mA. Samples in native sample buffer (10 μg protein, 15% glycerol, 62.5 mM Tris-Cl [pH 6.8], and 1% DOC) were size fractionated by electrophoresis for 60 min at 25 mA and then transferred to NC membrane, and the membrane was probed with rabbit anti-IRF3 MAb to detect IRF3 monomer and dimer.

### Indirect immunofluorescence assay.

An indirect immunofluorescence assay (IFA) was performed as previously described (57, 75–77, 86). HeLa cells were transfected with the indicated plasmid or vector for 24 h and then fixed in 4% paraformaldehyde (Beyotime Biotechnology). Cells were incubated with primary antibodies mouse anti-HA MAb (diluted 1:100) and rabbit anti-IRF3 MAb (diluted 1:100), followed by incubation with secondary antibodies Cy5-conjugated goat anti-rabbit IgG and FITC-conjugated goat anti-mouse IgG. Finally, cells were extensively washed with phosphate-buffered saline (PBS), and the nuclei were stained with Hoechst 33342 (Millipore Sigma). Samples were analyzed with confocal microscopy (SP8; Leica Microsystems, Buffalo Grove, IL, USA).

### Statistical analysis.

All data are shown as the mean values from biological replicates ± standard deviations (SD). Statistical analysis was performed using the two-tailed Student’s *t* test. Statistical significance is shown as follows: *, *P* < 0.05; **, *P* < 0.01; ***, *P* < 0.001; ****, *P* < 0.0001; ns, not statistically significant.
